# Impact of Empowering Leadership Training Through Flipped Learning Approach on the Well-Being of Nursing Staff and Residents in Long-Term Care Settings

**DOI:** 10.1155/jonm/5815383

**Published:** 2025-07-28

**Authors:** Tzu-Pei Yeh, Yen-Kuang Lin, Hsin-Yi Lu, Jacqueline Bloomfield, I-Hui Chen

**Affiliations:** ^1^School of Nursing, China Medical University, 100, Jingmao Rd., Sec. 1, Beitun District, Taichung 406040, Taiwan; ^2^Department of Nursing, China Medical University Hospital, 2, Yude Rd., North District, Taichung 404327, Taiwan; ^3^Graduate Institute of Athletics and Coaching Science, National Taiwan Sport University, 250, Wenhua 1st Rd., Guishan District, Taoyuan 333325, Taiwan; ^4^Community Healthcare Center, Taipei Medical University Hospital, 252, Wuxing St, Xinyi District, Taipei 11031, Taiwan; ^5^Sydney Nursing School, Faculty of Medicine and Health, The University of Sydney, Level 8 East, Susan Wakil Health Building, Camperdown, New South Wales 2006, Australia; ^6^School of Nursing, College of Nursing, Taipei Medical University, 250, Wuxing St, Xinyi District, Taipei 11031, Taiwan

**Keywords:** empowering leadership training, flipped learning approach, leader, long-term care, nursing staff, resident, well-being

## Abstract

**Objectives:** The benefits of empowering leadership have been documented, but few studies have examined its impacts on nursing staff job well-being and resident outcomes in long-term care (LTC) settings. In this study, we evaluated the effects of an empowering leadership training program, delivered via a flipped learning approach for LTC leaders, on nursing staff and resident well-being.

**Design:** A quasiexperimental pre-post design with a control group was used.

**Setting and Participants:** Participants were recruited from six residential facilities in central Taiwan, and included 80 staff (40 per group), 186 residents (intervention: 100; control: 86), and 21 leaders (10 in the intervention group and 11 in the control group).

**Intervention:** A 12-module intervention for LTC leaders comprised e-learning, face-to-face classes, and post-class assignments focused on empowering leadership. Each module lasted 2 h per week. The control group received an educational brochure covering the same topics.

**Methods:** Postintervention data were collected 3 months after the intervention. Staff measures included the Leader Empowerment Behavior Scale, Occupational Burnout Inventory Scale, Spreitzer's Empowerment Scale, Minnesota Job Satisfaction Questionnaire, and a single-item about job stress. Resident measures were the SF-36 health survey, WHO Quality of Life Questionnaire, and Customer Satisfaction Scale. Linear mixed models were employed to assess intervention effects. This study complied with the TREND checklist.

**Results:** Compared to controls, the intervention group demonstrated significant improvements over time in staff perceptions of empowering leader behaviors (*p* < 0.001), psychological empowerment (*p* < 0.001), job satisfaction (*p* < 0.001), and reduced burnout (*p* < 0.001). For residents, significant improvements in psychological health (*p* < 0.05), quality of life (*p* < 0.05), and care quality (*p* < 0.001) were detected in the intervention group.

**Conclusions and Implications:** The empowering leadership educational intervention for leaders using a flipped learning approach enhanced nursing staff job well-being and resident health outcomes in LTC settings. This intervention can provide a sustainable model for cultivating empowering leadership to optimize LTC workforce stability and resident care. Further research exploring mechanisms and long-term sustainability is warranted.

## 1. Introduction

Long-term care (LTC) settings present challenges for nursing staff, because of high workloads, emotional demands, and the need to care for residents with complex needs [1, 2]. Furthermore, LTC nursing staff often face staffing shortages, a lack of resources, and regulatory pressures [3]. Consequently, these factors contribute to high rates of burnout, job dissatisfaction, and turnover among LTC nursing staff [4], with estimates suggesting that annual turnover rates exceed 50% [5]. The job well-being of nursing staff in LTC settings has significant implications for resident outcomes and quality of care [5]. Studies have demonstrated associations of higher levels of nursing staff burnout and job dissatisfaction with adverse resident outcomes, including an increased risk of hospitalizations, the prevalence of pressure ulcers, use of physical restraints, and higher mortality rates [5, 6]. Conversely, a positive and supportive work environment for nursing staff is associated with better resident care quality and satisfaction [7].

Evidence highlights that leadership plays a pivotal role in shaping the culture and quality of care in LTC settings [8]. Effective leadership can foster a person-centered care environment that empowers staff, enhances teamwork, and ultimately improves resident outcomes [9]. Specifically, empowering leadership, a leadership style that emphasizes sharing decision-making, motivating employees, and promoting autonomy and their development, is associated with improved job satisfaction, reduced burnout, and better work performance [10, 11]. By fostering a sense of empowerment and psychological ownership among employees, empowering leaders can create a more positive, engaging, and psychologically safe work climate [12, 13], ultimately leading to better employee well-being and organizational outcomes [14]. While the benefits of empowering leadership have been documented, few studies have investigated its impacts on nursing staff job well-being and resident outcomes in LTC settings.

Empowering leadership is characterized by specific behaviors that share power with subordinates and enhance their self-determination and capabilities. Core elements of empowering leadership include (1) delegating authority and providing autonomy in decision-making; (2) fostering meaningful work by helping employees understand the significance of their contributions; (3) expressing confidence in employee capabilities and high performance expectations; (4) removing bureaucratic constraints that impede performance; and (5) encouraging participative decision-making, where employee input is solicited and valued [15–17].

Empowering leadership influences nursing staff outcomes through multiple mechanisms. First, by promoting autonomy and participation in decision-making, empowering leadership enhances staff members' sense of psychological ownership over their work and strengthens their intrinsic motivation [18, 19]. Second, when leaders demonstrate confidence in staff capabilities, they foster increased self-efficacy among the staff, which can reduce burnout and enhance job satisfaction [20]. Third, empowering leadership creates a supportive work environment that values staff contributions, potentially reducing job stress and enhancing psychological well-being [21].

For residents in LTC settings, the benefits of empowering leadership may manifest through several pathways. When the nursing staff experience greater psychological empowerment, they may develop an enhanced capacity to provide person-centered care that respects resident autonomy and preferences [22]. Empowered staff may also demonstrate greater initiative in promptly addressing resident needs and may be more likely to engage in proactive behaviors that enhance the quality of care [23]. In addition, reduced burnout among staff could result in more positive and meaningful interactions with residents, potentially enhancing residents' psychological well-being and quality of life [24].

Moreover, LTC facilities struggle with developing and sustaining strong leadership practices [25], possibly because of traditional leadership training programs for LTC leaders that often rely on passive, lecture-based methods that might not effectively translate into behavioral changes or organizational transformation [26]. The flipped learning approach, a learner-centered pedagogy that promotes active learning, engagement, and knowledge application to real-world scenarios [27], may equip LTC leaders with critical competencies to implement empowering leadership concepts as practical skills and behaviors within their facilities. Consequently, an empowered, engaged workforce guided by effective leaders could potentially enhance provision of high-quality, compassionate care prioritizing residents' physical and psychosocial well-being [28].

Previous evaluations of empowering leadership educational interventions showed promising results, although few were specifically conducted in LTC environments. The literature identifies education as a favorable intervention for developing leadership; however, only a limited number of empowering leadership educational interventions have been proposed. In healthcare settings, MacPhee et al. [29] and Dahinten et al. [30] assessed the impacts of an empowering leadership educational program on both nursing leaders from various organizations in Canada and their staff nurses, finding improvements in leaders' perceived use of empowering behaviors and staff outcomes. Similarly, Cougot et al. [31] implemented a program encompassing empowering leadership education, field training, and coaching for nursing and medical leaders, demonstrating reduced emotional exhaustion among personnel at a university hospital. Beyond healthcare, Martin et al. [32] evaluated the impacts of an empowering leadership educational program on business leaders from diverse professional organizations and showed positive effects on both employee and customer outcomes. However, existing interventions have predominantly utilized traditional didactic teaching methods rather than innovative approaches. In addition, while leadership interventions in LTC have been examined, including coaching-focused approaches that have demonstrated positive effects on staff knowledge use and job satisfaction [33], significant knowledge gaps persist. Specifically, limited research has investigated empowering leadership as a distinct theoretical construct within LTC contexts, employed innovative pedagogical methods such as flipped learning, or assessed cascading effects on resident outcomes. The current study addresses these gaps by implementing a flipped learning approach to empowering leadership training and examining both staff and resident outcomes in LTC settings.

In this study, we aimed to evaluate the impacts of an empowering leadership training program delivered through a flipped learning approach for LTC leaders on staff and resident well-being outcomes. We hypothesized the following:

• Hypothesis 1: Staff whose leaders were trained using a flipped-learning approach will have higher perceived empowerment behaviors, lower occupational burnout, higher psychological empowerment, higher job satisfaction, and lower job stress than staff whose leaders were trained using a conventional learning approach.
• Hypothesis 2: Residents cared for by staff whose leaders were trained using a flipped-learning approach will have better physical and mental health, a higher quality of life, and higher customer satisfaction than those cared for by staff whose leaders were trained using conventional approaches.

## 2. Methods

### 2.1. Design

This quasiexperimental pre-post study with a control group was conducted across six nonprofit residential LTC facilities in central Taiwan from August 2018 to May 2019. Facility size ranged from 110 to 200 beds (with a mean occupancy rate of 92%), and comprised two public and four private organizations. Facilities were nonrandomly assigned to the intervention (*n* = 2) or control (*n* = 4) group based on leadership willingness to participate. No structural or staffing ratio changes occurred in the facilities during the intervention period. This study was approved by the Research Ethics Committee of China Medical University Hospital (CRREC-105-002) and was guided by the Transparent Reporting of Evaluations with Nonrandomized Designs [34].

### 2.2. Participants

**Figure 1:**
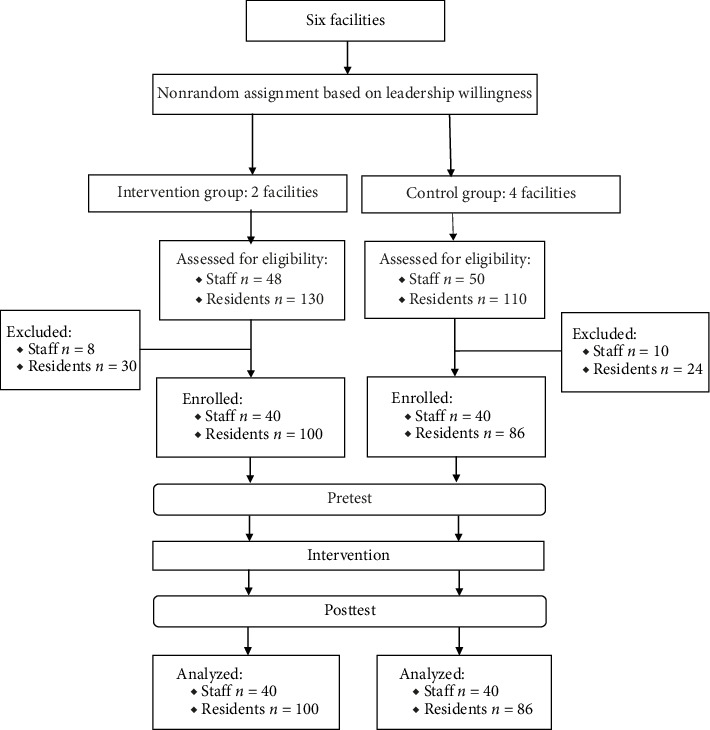
Study flowchart.

Staff inclusion criteria consisted of (1) being a registered nurse, licensed practical nurse, or nursing assistant; (2) having served in the current facility for, at least, 3 months; (3) being ≥ 20 years old; and (4) giving informed consent to participate. Resident inclusion criteria were (1) having resided in the current facility for at least 3 months to allow adequate adjustment to the care environment, consistent with LTC transition research [35]; (2) having a Mini-Mental State Examination score of ≥ 24 to ensure sufficient cognitive capacity for informed consent and study participation [36]; (3) having no severe hearing impairment to facilitate effective communication during data collection; (4) not being terminally ill to reduce participant burden during end-of-life periods and ensure study completion feasibility; and (5) providing written informed consent to participate. A priori power analysis using the sjstats package in R was conducted to determine required sample sizes. Because of limited published data on effect sizes and intraclass correlation coefficients (ICC) for similar nursing interventions in LTC settings, parameters were derived from comparable studies in healthcare and organization-related research. For staff outcomes, we assumed Cohen's *d* = 0.7 and ICC = 0.2 [37, 38]. For resident outcomes, we conservatively assumed Cohen's *d* = 0.5 and ICC = 0.4 [39–41]. With *α* = 0.05 and 80% power, the analysis indicated minimum required samples of 79 staff and 179 residents. In the intervention group, 48 staff were assessed for eligibility, of whom 40 were enrolled. Among residents, 130 were assessed and 100 were enrolled. In the control group, 50 staff were assessed and 40 were enrolled, while 110 residents were assessed and 86 were enrolled (Figure 1).

### 2.3. Empowering Leadership Training Through Flipped Learning

The intervention, developed by the research team based on the theory of empowering behavior leadership [10, 11], comprised 12 modules delivered through a combination of e-learning, face-to-face classes, and post-class assignments. The intervention exclusively targeted leaders, comprising 10 nursing directors, supervisors, or heads with a minimum of 6 months' experience in their current role. The control group included 11 leaders with similar positions and experience requirements. Each module was 2 h per week. The modules specifically addressed the five core elements of empowering leadership: delegating authority, fostering meaningful work, expressing confidence in staff capabilities, removing bureaucratic constraints, and encouraging participative decision-making. E-learning sessions, delivered through 60-min videos, focused on empowerment concepts and their practical applications, with specific topics outlined in Supporting Table 1. Following each e-learning session, leaders attended in-class sessions of 1 h duration. Leaders were divided into two groups for discussions, practice, and instructor-guided inquiry. Case studies were presented, with leaders subdivided for practice discussions and proposed solutions, followed by real-time feedback from instructors. Post-class activities reinforced the concepts covered in sessions through reflections and tailored assignments to facilitate applied knowledge translation.

### 2.4. Empowering Leadership Training Through Conventional Learning

Leaders in the control group received weekly resource material on empowerment and empowering behaviors, covering the same topics and content as the intervention group. The research team maintained regular contact with the control group to provide reminders to read the material; however, no additional support or intervention was offered.

### 2.5. Measures

Data on staff and residents were collected at two points: at the baseline upon enrollment and postintervention. Postintervention data were collected 3 months after intervention completion, adhering to baseline procedures. This 3-month follow-up period was selected based on previous leadership intervention studies which indicated that changes in leadership behaviors typically require at least 8–12 weeks to translate into measurable outcomes [42, 43], making 3 months a suitable timeframe, while still minimizing confounding from staff turnover or organizational changes. Each data collection period—baseline and postintervention—spanned 1 month.

### 2.6. Staff Measures

The Leader Empowerment Behavior Scale was modified to measure staff perceptions of their leader's empowering behaviors across five subscales: extending work significance, being involved in decision-making, showing confidence, encouraging goal achievement, and allowing autonomy [44, 45]. A sample item is “My leader helps me understand that I am a member of the team.” Responses use a seven-point Likert scale, with higher scores implying higher perceptions by the staff of their leader's empowering behaviors. Cronbach's α in this study was 0.85, and the test–retest reliability using Pearson's coefficient was 0.71.

The 21-item Occupational Burnout Inventory Scale measures burnout across four subscales: personal, work-related, client-related, and over-commitment to work [46]. A sample item is “I feel physically exhausted because of my work.” Responses use a five-point Likert scale, with higher scores signifying higher burnout in the workplace. Cronbach's α was 0.80, and the test–retest reliability was 0.78.

The 12-item Spreitzer's Empowerment Scale measures psychological empowerment across four subscales: meaning, competence, self-determination, and impact [47, 48]. A sample item is “The work I do is meaningful to me.” Responses use a five-point Likert scale, with higher scores indicating higher levels of psychological empowerment. Cronbach's α was 0.87, and the test–retest reliability was 0.82.

The 20-item Minnesota Satisfaction Questionnaire Short Form measures job satisfaction [49, 50]. A sample item is “On my present job, this is how I feel about the chance to do different things from time to time.” Responses use a five-point Likert scale, with higher scores indicating higher job satisfaction. Cronbach's α was 0.88, and the test–retest reliability was 0.84.

Job stress was evaluated using a single five-point Likert item “Do you often feel very stressed at work?”, with a higher score implying higher job stress. Single-item global stress measures demonstrate acceptable construct validity and are commonly used in occupational health research when survey space is limited [51, 52].

A questionnaire collected staff characteristics including age, gender, marital status, educational level, license type, years of caring experience, and years of employment at the current facility.

### 2.7. Resident Measures

The Short Form-36 (SF-36) Health Survey measures physical and mental health across eight domains: physical functioning, social functioning, role limitations because of physical health, pain, emotional well-being, role limitations because of emotional problems, energy, and general health [53]. A sample item is “During the past 4 weeks, how much of the time have you felt calm and peaceful?” Scores range 0–100, with higher scores indicating a better health status. Cronbach's α values in this study were 0.78–0.90, and values of the test–retest reliability using Pearson's coefficient were 0.86–0.91. One self-reported health transition item was excluded from the analyses as it measures perceived health change over time rather than current health status and is not incorporated into the standard SF-36 domain scoring system.

The World Health Organization Quality of Life Questionnaire (WHOQOL-BREF) measures quality of life across four subscales: physical health, psychological health, social relationships, and environmental health [54]. A sample item is “How satisfied are you with your ability to perform your daily living activities?” Responses use a five-point Likert scale, with higher scores implying a better quality of life. Cronbach's α values in this study were 0.80–0.88, and values of the test–retest reliability were 0.90–0.95.

The 25-item Customer Satisfaction Scale measures care quality across four subscales: comfort/cleanliness, nursing care, food services, and facility care/services [55, 56]. A sample item is “How satisfied are you with the cleanliness of the facility?” Responses use a five-point Likert scale, with higher scores indicating higher care quality. Cronbach's α was 0.94, and the test–retest reliability was 0.75.

Resident characteristics including age, gender, marital status, educational level, number of chronic diseases, number of children, self-reported financial satisfaction, years of residing in the current facility, activities of daily living (ADLs) [57], and instrumental ADLs (IADLs) [58] were collected via a questionnaire. Higher ADL and IADL scores indicated greater disability.

### 2.8. Data Collection

Following approval from the Research Ethics Committee of China Medical University Hospital and agreement from each facility, information sessions were conducted to inform and invite staff and residents to participate in the study. To ensure anonymity while linking pre- and postintervention responses, participants were instructed to write a serial number we provided on the front page of each survey. Staff were given a designated time during work hours to independently complete the questionnaires. For resident participants, trained interviewers administered the questionnaires using a person-centered approach. Recognizing the potential burden on residents, especially those with functional limitations, the interviews were conducted in multiple shorter sessions as needed, allowing for breaks and accommodating individual preferences and energy levels. Resident demographics were extracted from their health records to ensure accuracy and minimize participant burden.

### 2.9. Ethical Considerations

This study received approval from the Research Ethics Committee of China Medical University Hospital (approval no.: CRREC-105-002). Prior to participant screening, the research team conducted thorough briefing sessions to explain the study's purpose, methodology, and potential risks and benefits. Written informed consent was obtained from all participants, with particular attention given to ensuring comprehension among residents who may have had functional limitations. Participants were assured of data confidentiality and their right to withdraw from the study at any time without consequences, emphasizing the voluntary nature of participation. All data were de-identified and securely stored, with access restricted to authorize research personnel only.

### 2.10. Data Analysis

Baseline variables were analyzed using means and standard deviations (SDs) for continuous variables and frequencies and percentages for categorical variables. Normality was assessed via frequency histograms. Chi-squared and *t*-tests examined between-group differences at the baseline. All leaders who initially agreed to participate completed the entire training program with full attendance at all sessions, yielding complete data for the leadership component. Importantly, participation in all training modules was entirely voluntary, with no mandatory attendance requirements imposed by facility management or the research team. The 100% completion rate reflects genuine engagement and commitment from leaders who volunteered to participate in the study. Consequently, our analysis represented both intent-to-treat and per-protocol approaches, as they were identical in this study. The analysis focused on outcomes at the staff and resident levels, with leaders' complete participation serving as the implementation vehicle for the intervention at each facility. To account for the hierarchical structure of the data, a three-level linear mixed-effects model was employed. Level 1 represented repeated measurements (pre- and postintervention) nested within individual participants (Level 2: staff or residents), who were in turn nested within facilities (Level 3). This model enabled estimates of both group-level and individual-level intervention effects over time, with random effects specified to account for variability at each level. The intervention effect was evaluated by examining the significance of the group × time interaction term, which indicated differential change over time between the intervention and control groups. Following best practices for statistical control [59], both unadjusted and adjusted models were fitted, with adjusted models including baseline covariates that showed significant between-group differences to assess their impact on main effects. Random effects were specified at the facility level (staff/residents within a facility) and residual level to account for clustering and within-person variability. The variance components of these random effects were estimated to quantify between-facility and within-cluster variability. Significance was set at *p* < 0.05. Analyses utilized SPSS vers. 22 (SPSS Inc) and SAS vers. 9.4 (SAS Institute).

## 3. Results

Compared to the control group, staff in the intervention group were younger, had a higher proportion of married individuals, and had a larger percentage of nursing assistants, but they had less caring experience and shorter employment durations at the current facility (Table 1). Differences in age and years of caring experience between the two groups were statistically significant at the baseline.

Residents in the intervention group were older, with more chronic diseases, more children, longer facility residence, and greater functional impairment (higher ADL and IADL scores) but lower financial satisfaction compared to the control group (Table 2). Statistically significant differences between the two groups at the baseline were observed for age, marital status, educational level, children, satisfaction with their financial situation, residence duration, ADLs, and IADLs.

### 3.1. Effects of the Intervention on Staff

The intervention demonstrated significant effects on multiple staff outcomes as shown in Tables 3 and 4, with detailed interaction effects for all subscales presented in Supporting Table 2 and changes in mean outcome scores visualized in Supporting Figure 1. The intervention group showed improvement in staff perceptions of empowering leadership behaviors, with mean scores increasing from 139.6 (SD = 23.1) at the baseline to 162.3 (SD = 12.2) at follow-up, while control group scores declined from 138.6 (SD = 20.1) to 131.5 (SD = 15.8). The group × time interaction was highly significant (*B* = −29.8, 95% confidence interval (CI) [−34.1, −25.5], *p* < 0.001), indicating the intervention's effectiveness in enhancing staff perceptions of empowering leadership behaviors. All five dimensions of empowering leadership behaviors showed significant improvements (*p* < 0.001 for all dimensions).

The intervention produced substantial reductions in occupational burnout, with mean scores decreasing from 38.5 (SD = 12.4) to 25.2 (SD = 10.1) in the intervention group, while they increased from 33.9 (SD = 10.1) to 39.0 (SD = 9.3) in the control group. The group × time interaction was significant (*B* = 18.4, 95% CI [16.3, 20.5], *p* < 0.001), suggesting the intervention's effectiveness in reducing burnout. All dimensions of burnout (personal burnout, work-related burnout, client-related burnout, and overcommitment to work) showed significant improvements (all *p* < 0.001).

Psychological empowerment scores in the intervention group increased from 43.6 (SD = 5.8) to 49.0 (SD = 3.9), while control group scores decreased from 43.4 (SD = 5.4) to 41.4 (SD = 5.0). The group × time interaction effect was significant (*B* = −7.5, 95% CI [−8.7, −6.2], *p* < 0.001), demonstrating the intervention's positive impact on psychological empowerment. All four dimensions of psychological empowerment (meaning, competence, self-determination, and impact) demonstrated significant improvements (all *p* < 0.001).

Job satisfaction markedly improved in the intervention group, with mean scores increasing from 70.0 (SD = 8.8) to 80.3 (SD = 6.9), while they decreased in the control group from 68.3 (SD = 8.8) to 64.0 (SD = 6.5). The group × time interaction was significant (*B* = −14.6, 95% CI [−16.7, −12.5], *p* < 0.001), demonstrating the intervention's positive effect on job satisfaction.

In contrast to other outcomes, job stress showed no significant change after the intervention. Mean values of job stress remained relatively stable in both groups (intervention: 3.3 to 3.1; control: 3.1 to 3.0), with a nonsignificant group × time interaction (*B* = 0.1, 95% CI [−0.1, 0.2], *p*=0.53).

The linear mixed-effects model accounted for the nested data structure (staff within facilities) and controlled for years of caring experience and age. Comparison between unadjusted and adjusted models showed consistent intervention effects. Years of caring experience showed a small but significant association with psychological empowerment (*B* = 0.2, 95% CI [0.01, 0.4], *p* < 0.05), while neither years of experience nor age significantly influenced other outcome measures. The model's random effects components revealed substantial between-staff variability within facilities, with the largest variance components observed for staff perceptions of empowering leadership behaviors (variance = 289.3) and burnout (variance = 98.7). The considerable within-facility variance components (ranging from 0.1 to 46.7 across outcomes) further validated the appropriateness of using a linear mixed-effects modeling approach to account for the hierarchical data structure. In conclusion, hypothesis 1 was mostly supported.

### 3.2. Effects of the Intervention on Residents

The intervention demonstrated significant effects on multiple resident outcomes as shown in Tables 5, 6, and 7, with detailed interaction effects for all subscales presented in Supporting Table 2 and changes in mean outcome scores visualized in Supporting Figures 2 and 3. The intervention group showed improvement in mental health regarding role limitations because of emotional problems, with mean scores increasing from 27.0 (SD = 40.1) at the baseline to 41.3 (SD = 39.9) at follow-up, while the control group showed a minimal change from 69.8 (SD = 42.4) to 65.5 (SD = 42.6). The group × time interaction was significant (*B* = −18.3, 95% CI [−34.0, −2.7], *p*=0.02). Similarly, mental health social functioning scores improved in the intervention group from 58.4 (SD = 22.8) to 67.4 (SD = 20.0), while declining in the control group from 83.4 (SD = 17.6) to 79.1 (SD = 21.0), with a significant group × time interaction (*B* = −13.2, 95% CI [−21.1, −5.4], *p*=0.001). Improvement was also observed in general health perceptions scores, which increased from 46.0 (SD = 13.6) to 57.3 (SD = 11.6) in the intervention group, while the control group showed minimal change from 62.2 (SD = 24.6) to 57.2 (SD = 21.8). The group × time interaction was significant (*B* = −16.2, 95% CI [−23.0, −9.3], *p* < 0.001). The intervention group also showed a trend toward improvement in energy (*p*=0.06), with mean scores increasing from 49.6 (SD = 13.1) to 54.0 (SD = 12.0), although this did not reach statistical significance.

Quality of life outcomes showed improvements across all domains in the intervention group compared to the control group. The linear mixed-effects model revealed significant group × time interactions for physical health (*B* = −1.9, 95% CI [−2.7, −1.1], *p* < 0.001), psychological health (*B* = −1.4, 95% CI [−2.2, −0.6], *p* < 0.01), social relationships (*B* = −1.9, 95% CI [−2.7, −1.1], *p* < 0.001), and environmental health (*B* = −1.8, 95% CI [−2.5, −1.2], *p* < 0.001).

Customer satisfaction improved in the intervention group, with total scores increasing from 94.8 (SD = 14.0) to 104.3 (SD = 10.4), while decreasing in the control group from 96.5 (SD = 10.7) to 90.6 (SD = 9.3). The group × time interaction was significant (*B* = −15.3, 95% CI [−19.6, −11.0], *p* < 0.001). All dimensions of customer satisfaction—comfort and cleanliness, nursing care, food services, and facility care and services—showed significant improvements (all *p* < 0.001).

The linear mixed-effects models accounted for the nested data structure (residents within facilities) and controlled for age, marital status, educational level, number of children, financial satisfaction, years residing in the facility, ADLs, and IADLs. Comparison between unadjusted and adjusted models showed that most intervention effects remained consistent. The models' random effects components revealed substantial between-resident variability within facilities, with the largest variance components observed for physical functioning (648.5) and role limitations because of physical health (1340.4) and customer satisfaction (111.9). The considerable within-facility variance components further validated the appropriateness of using a linear mixed-effects modeling approach to account for the hierarchical data structure. In summary, hypothesis 2 was largely supported.

## 4. Discussion

To our knowledge, this is the first study to examine cascade effects of structured empowering leadership education on both nursing staff and resident outcomes in LTC facilities using a multilevel design with multisource data collection. While previous evidence suggested that positive leadership can reduce adverse patient outcomes, those conclusions were indirectly drawn from nurse reports [1, 58, 60], rather than directly from patient data.

Workforce challenges represent one of the most formidable issues facing LTC facilities, with unhealthy work environments diminishing nursing staff quality of life and driving turnover [61–63]. This study demonstrated that staff working under leaders trained via the flipped learning approach experienced a constellation of favorable outcomes—increased staff perceptions of empowering leadership behaviors, elevated psychological empowerment, reduced job burnout, and greater job satisfaction. The flipped learning approach appears to enhance engagement skills, interpersonal abilities, problem-solving capacities, and communication competencies among leaders [64], which facilitate empowering behaviors toward staff. Empowering leaders can establish conditions for psychological empowerment by implementing empowerment structures and an autonomy-encouraging culture (e.g., providing feedback and rewards) [13]. Moreover, empowered leaders have the potential to facilitate interdisciplinary cooperation. Their behaviors directly and comprehensively influence their staff's sense of effectiveness, meaning, influence, and autonomy [65], contributing to improved work-related outcomes among staff members. These findings reinforce established linkages between empowering leadership and improved employee well-being over time [66, 67]. Hence, using the flipped learning approach to foster and enhance empowering leadership may be a worthwhile investment for nursing leaders in LTC facilities.

The observed improvements in staff outcomes aligned with the theoretical mechanisms of empowering leadership outlined earlier. The intervention appeared to have successfully activated key pathways through which empowering leadership influences staff well-being. First, as leaders implemented behaviors that promoted autonomy and participative decision-making, staff reported higher levels of psychological empowerment, particularly in the self-determination dimension. This supported the proposed mechanism that empowering leadership enhances staff's sense of psychological ownership and intrinsic motivation through autonomous decision-making opportunities. Second, the significant reduction in burnout and improvement in job satisfaction suggested that leaders' expressions of confidence in staff capabilities effectively enhanced staff self-efficacy, as theorized. Third, the creation of a supportive work environment that valued staff contributions likely contributed to the observed improvements in staff perceptions of meaning in their work. These findings reinforced the multidimensional nature of empowering leadership effects on staff outcomes proposed in previous research [15, 16].

The intervention did not significantly impact the staff-reported job stress levels, which contrasts with literature suggesting empowered nurses experience reduced job stress [68]. This finding may reflect contextual factors specific to Taiwan's healthcare environment. In high power-distance cultures [69], increased autonomy from empowering leadership may initially create adaptation challenges as staff members adjust to enhanced decision-making responsibilities [31]. In addition, Taiwan's ongoing LTC nursing shortage contributes to persistently high job demands [70, 71], which may moderate the stress-reducing effects of empowerment. These contextual factors are important to consider when implementing empowering leadership interventions in similar healthcare settings.

Our findings regarding resident outcomes also support the proposed mechanisms through which empowering leadership affects care recipients. Improvements in residents' mental health status, quality of life, and perceived care quality suggested that the empowering leadership intervention successfully cascaded from leaders to staff and ultimately to residents, as hypothesized. Empowered staff likely demonstrated greater initiatives in addressing resident needs and engaged in more personalized, autonomous care practices [23]. The significant improvements in resident psychological health and social functioning, in particular, may reflect enhanced quality of staff-resident interactions resulting from reduced staff burnout [24]. These findings extend previous research on empowering leadership by empirically demonstrating its benefits beyond the immediate leader-staff relationship to include positive impacts on care recipients, addressing an important gap in the leadership literature in healthcare settings.

Providing high-quality care supporting the well-being of residents in LTC settings is a critical priority [72]. This study found that residents in the intervention group had better mental health status, elevated quality of life, and better perceived care quality. These residents likely perceived empowering behaviors from leaders and staff, fostering feelings of respect and self-confidence that activated self-care management participation [73] and a better mental health functional status, ultimately enhancing their quality of life. Moreover, the empowered staff likely contributed to improvements in care quality through several mechanisms. First, increased self-efficacy, engagement, and job satisfaction among empowered staff may have enhanced their motivation and commitment to providing high-quality care [10, 11], resulting in better management of residents' health needs. Second, reduced job burnout could have led to improved staff-resident interactions, fostering a more positive and supportive care environment [74]. Third, psychologically empowered staff may have been more proactive in identifying and addressing resident concerns, elevating care quality and overall resident quality of life [75]. Person-centered care models, an increasingly advocated approach in current LTC settings, emphasize resident empowerment as a core element [76, 77]. Implementation of such models through empowered nursing leadership and staff, as demonstrated in this intervention, may enable residents to experience a sense of control, and their needs may be better met. Thus, the present intervention may facilitate the adoption of person-centered care principles in LTC facilities by fostering resident empowerment. On the other hand, the intervention did not demonstrate promising impacts on resident physical health at the 3-month postintervention follow-up. A longer timeframe may be needed to detect changes in these outcomes. While staff perceived leader support, translating empowerment perceptions into work effectiveness, such as increased comprehensive geriatric assessments to reduce pain, may require more time [78]. In addition, these outcomes might not have been primary intervention targets, resulting in insufficient direct targeting during implementation.

This study's methodological strengths include its multilevel design examining outcomes across staff and residents, and the inclusion of direct resident measures rather than relying solely on staff-reported proxies. Multisource data collection reduces common method variance. Contextually, implementation in LTC settings—characterized by complex care needs and workforce challenges [77]—provides valuable insights for healthcare administrators seeking evidence-based leadership development approaches. However, several limitations warrant consideration. Because of the quasiexperimental design, several internal validity threats may limit the findings' explanations. The lack of randomization introduces potential selection bias and confounding variables that could have influenced the observed outcomes. Our study design limits conclusions about comparative effectiveness of different educational approaches, as our control group received passive resource materials rather than structured conventional training. Therefore, our findings demonstrate the effectiveness of structured empowering leadership education rather than the superiority of any specific educational methodology. Generalizability may be limited by the small sample size from a few facilities in Taiwan and the voluntary nature of participation, as self-selected motivated participants may not be representative of the broader healthcare populations. The relatively small number of leaders restricted examination of potential moderating effects of leader characteristics (experience, formal education, prior leadership training, and preintervention leadership style), preventing the determination of optimal intervention conditions and potentially introducing bias in results. The study also relied on self-reported outcomes which are subject to common method variance bias. A notable limitation is the absence of a formal process evaluation to understand implementation mechanisms. As Biron and Karanika-Murray [79] argued, process evaluation can provide critical insights into why, how, and under what circumstances interventions are effective in organizational settings. Our study would have benefited from a systematic assessment of processes of implementation fidelity, participant engagement with the flipped learning components, contextual factors influencing uptake, and leadership behavioral changes. This limitation stems from time and funding constraints that restricted our ability to collect such supplementary qualitative and quantitative implementation data. Finally, the 3-month follow-up period after the intervention may have been too short to determine sustained effects over a longer duration, such as 6 months or 1 year.

## 5. Conclusions and Implications

The flipped learning empowering leadership intervention demonstrated promising impacts on several key outcomes. Staff in the intervention group reported improved perceptions of empowering leadership behaviors, increased psychological empowerment, enhanced job satisfaction, and reduced job burnout. Concurrently, residents experienced improvements in health, quality of life, and care quality. Our study advances the understanding of empowering leadership in LTC settings by demonstrating the effectiveness of structured leadership education and providing evidence for theorized mechanisms through which empowering leadership influences both staff and resident outcomes. The findings suggest that developing empowering leadership capacities among LTC leaders creates a positive cascade effect, enhancing staff psychological empowerment and job satisfaction, which subsequently improves resident care quality and well-being. Policymakers and LTC administrators should consider investing in leadership development programs that specifically target the core components of empowering leadership—autonomy support, meaningful work, confidence building, barrier removal, and participative decision-making—to address persistent challenges of staff dissatisfaction and turnover. Regulatory bodies might explore strategies to promote and support implementation of evidence-based leadership development programs in LTC facilities, recognizing the multilevel benefits for both staff and residents. Future research should investigate the underlying mechanisms by which leadership interventions influence outcomes, exploring potential mediators such as person-centered care practices or staff resilience. In addition, future comparative effectiveness research examining different educational methodologies for empowering leadership training is needed to optimize intervention delivery in healthcare settings. Robust longitudinal experimental studies with larger samples of leaders across more facilities are also needed to assess the long-term sustainability of intervention effects and identify factors contributing to the maintenance of empowering leadership behaviors.

## Figures and Tables

**Table 1:** Baseline information of nursing staff in the intervention (*N* = 40) and control (*N* = 40) groups.

Variable	Intervention		Control		*t*/*x*^2^	*p* value
	Mean (SD)	*n* (%)	Mean (SD)	*n* (%)		
Age (years)	35.8 (9.1)		45.1 (12.1)		−3.9	< 0.001
Female		39.0 (97.5)		40.0 (100.0)	1.0	1.0
Marital status					0.8	0.371
Married		22.0 (55.0)		18.0 (45.0)		
Unmarried		18.0 (45.0)		22.0 (55.0)		
Educational level					0.35	1.0
University/college		39.0 (97.5)		38.0 (95.0)		
Graduate school		1.0 (2.5)		2.0 (5.0)		
Licensure					0.27	0.606
Nurse		9.0 (22.5)		11.0 (27.5)		
Assistant		31.0 (77.5)		29.0 (72.5)		
Years of caring experience	12.2 (8.0)		19.0 (9.7)		−3.4	0.001
Years of employment with this facility	4.9 (4.9)		5.8 (5.2)		−0.8	0.441

Abbreviation: SD, standard deviation.

**Table 2:** Baseline information of residents in the intervention (*N* = 100) and control (*N* = 86) groups.

Variable	Intervention		Control		*t*/*x*^2^	*p* value
	Mean (SD)	*n* (%)	Mean (SD)	*n* (%)		
Age (years)	78.6 (8.7)		65.8 (14.8)		7.3	<0.001
Gender					0.3	0.601
Female		45.0 (45.0)		42.0 (48.8)		
Male		55.0 (55.0)		44.0 (51.2)		
Marital status					7.8	0.005
Married		40.0 (40.0)		18.0 (20.9)		
Unmarried		60.0 (60.0)		68.0 (79.1)		
Educational level					12.5	0.006
Elementary school		46.0 (46.0)		32.0 (37.2)		
Junior high school		18.0 (18.0)		11.0 (12.8)		
Senior high school		27.0 (27.0)		18.0 (20.9)		
University/college above		9.0 (9.0)		25.0 (29.1)		
Number of chronic diseases	2.0 (1.2)		1.8 (1.9)		0.8	0.455
Number of children	0.7 (0.5)		0.5 (0.5)		3.0	0.003
Satisfied with own financial situation	2.7 (0.8)		3.6 (0.9)		−7.2	<0.001
Years of residing in this facility	4.7 (4.7)		3.2 (2.9)		12.2.5	0.014
ADLs	16.1 (5.6)		6.9 (2.3)		15.0	<0.001
IADLs	27.5 (7.7)		11.6 (4.2)		17.7	<0.001

Abbreviations: ADLs, activities of daily living; IADLs, instrumental activities of daily living; SD, standard deviation.

**Table 3:** Nursing staff outcomes in the intervention (*N* = 40) and control (*N* = 40) groups.

Outcome	Baseline		Follow-up		*p* value^a^
	Intervention mean (SD)	Control mean (SD)	Intervention mean (SD)	Control mean (SD)	
Perceptions of empowering behavior leadership scale	139.6 (23.1)	138.6 (20.1)	162.3 (12.2)	131.5 (15.8)	< 0.001
Enhancing the meaningfulness of work subscale	32.5 (5.8)	32.2 (5.5)	38.1 (2.6)	30.2 (4.4)	< 0.001
Fostering participation in decision-making subscale	26.8 (5.8)	27.1 (3.8)	31.4 (2.8)	25.9 (2.8)	< 0.001
Facilitating goal accomplishment subscale	24.0 (4.3)	24.0 (4.3)	27.9 (3.7)	22.7 (3.8)	< 0.001
Expressing confidence in high performance subscale	31.3 (5.3)	30.5 (5.1)	36.4 (2.6)	29.3 (4.3)	< 0.001
Providing autonomy from bureaucratic constraints subscale	24.9 (5.2)	24.9 (4.3)	28.5 (3.6)	23.4 (3.8)	< 0.001
Occupational burnout inventory scale	38.5 (12.4)	33.9 (10.1)	25.2 (10.1)	39.0 (9.3)	< 0.001
Personal burnout subscale	11.0 (3.8)	9.7 (3.7)	7.1 (3.8)	11.0 (3.0)	< 0.001
Work-related burnout subscale	9.9 (4.2)	8.6 (3.7)	6.7 (3.5)	10.1 (3.7)	< 0.001
Client-related burnout subscale	7.3 (4.8)	6.8 (4.3)	4.8 (3.6)	7.8 (4.7)	< 0.001
Over-commitment to work subscale	10.3 (3.9)	8.9 (3.2)	6.7 (3.3)	10.2 (3.5)	< 0.001
Psychological empowerment scale	43.6 (5.8)	43.4 (5.4)	49.0 (3.9)	41.4 (5.0)	< 0.001
Meaning subscale	11.5 (1.8)	11.3 (2.0)	13.1 (1.2)	10.8 (1.8)	< 0.001
Competence subscale	12.0 (2.0)	11.9 (1.7)	13.5 (1.4)	11.4 (1.8)	< 0.001
Self-determination subscale	11.5 (2.1)	11.7 (1.8)	13.0 (1.4)	11.0 (1.8)	< 0.001
Impact subscale	8.6 (2.1)	8.5 (2.3)	9.5 (1.9)	8.2 (2.3)	< 0.001
Job satisfaction	70.0 (8.8)	68.3 (8.8)	80.3 (6.9)	64.0 (6.5)	< 0.001
Job stress	3.3 (0.7)	3.1 (0.7)	3.1 (0.6)	3.0 (0.5)	0.53

Abbreviation: SD, standard deviation.

^a^The *p* value corresponds to the group × time interaction effect from the linear mixed model controlling for age and years of caring experience (see Table 4 for complete model details).

**Table 4:** Linear mixed-effects model results for staff outcomes.

Parameter	Leader empowerment behavior		Burnout		Psychological empowerment		Job satisfaction		Job stress	
	Unadjusted	Adjusted	Unadjusted	Adjusted	Unadjusted	Adjusted	Unadjusted	Adjusted	Unadjusted	Adjusted
*Fixed effects*										
Intercept	131.5 (125.7, 137.2)^∗∗∗^	139.6 (120.0, 159.2)^∗∗∗^	39.0 (35.7, 42.3)^∗∗∗^	44.0 (32.7, 55.3)^∗∗∗^	41.4 (39.8, 43.0)^∗∗∗^	37.0 (32.0, 42.1)^∗∗∗^	64.0 (61.5, 66.4)^∗∗∗^	56.5 (48.3, 64.7)^∗∗∗^	3.0 (2.8, 3.2)^∗∗∗^	3.2 (2.5, 3.8)^∗∗∗^
Time	7.1 (4.0, 10.1)^∗∗∗^	7.1 (4.0, 10.1)^∗∗∗^	−5.1 (−6.6, −3.6)^∗∗∗^	−5.1 (−6.6, −3.6)^∗∗∗^	2.0 (1.1, 2.9)^∗∗∗^	2.0 (1.1, 2.9)^∗∗∗^	4.3 (2.8, 5.8)^∗∗∗^	4.3 (2.8, 5.8)^∗∗∗^	0.1 (0.01, 0.2)^∗^	0.1 (0.01, 0.2)^∗^
Group	30.8 (22.7, 39.0)^∗∗∗^	30.4 (21.6, 39.3)^∗∗∗^	−13.8 (−18.5, −9.1)^∗∗∗^	−13.9 (−18.9, −8.8)^∗∗∗^	7.6 (5.3, 9.9)^∗∗∗^	9.1 (6.8, 11.4)^∗∗∗^	16.3 (12.8, 19.8)^∗∗∗^	17.8 (14.1, 21.6)^∗∗∗^	0.1 (−0.2, 0.4)	0.1 (−0.2, 0.4)
Group × time	−29.8 (−34.1, −25.5)^∗∗∗^	−29.8 (−34.1, −25.5)^∗∗∗^	18.4 (16.3, 20.5)^∗∗∗^	18.4 (16.3, 20.5)^∗∗∗^	−7.5 (−8.7, −6.2)^∗∗∗^	−7.5 (−8.7, −6.2)^∗∗∗^	−14.6 (−16.7, −12.5)^∗∗∗^	−14.6 (−16.7, −12.5)^∗∗∗^	0.1 (−0.1, 0.2)	0.1 (−0.1, 0.2)
Years of caring experience		0.5 (−0.3, 1.2)		0.4 (−0.1, 0.8)		0.2 (0.01, 0.4)^∗^		−0.02 (−0.3, 0.3)		0.01 (−0.02, 0.04)
Age		−0.4 (−1.0, 0.3)		−0.3 (−0.6, 0.1)		0.01 (−0.1, 0.2)		0.2 (−0.1, 0.4)		−0.01 (−0.03, 0.01)

*Random effects: covariance estimates*										
Staff within facility (intercept)	287.5	289.3	99.6	98.7	21.6	18.5	50.2	48.6	0.3	0.3
Residual	46.7	46.7	11.2	11.2	4.2	4.2	11.3	11.3	0.1	0.1

*Note:* Values are parameter estimates with 95% confidence intervals in parentheses. Sample sizes: Number of facilities = 6; number of staff = 80; number of observations = 160 (80 staff × 2 time points).

^∗^ *p* < 0.05.

^∗∗^ *p* < 0.01.

^∗∗∗^ *p* < 0.001.

**Table 5:** Resident outcomes in the intervention (*N* = 100) and control (*N* = 86) groups.

Outcome	Baseline		Follow-up		*p* value^a^
	Intervention mean (SD)	Control mean (SD)	Intervention mean (SD)	Control mean (SD)	
SF-36 physical functioning	22.7 (31.3)	69.8 (27.4)	24.5 (31.2)	69.1 (27.5)	0.70
SF-36 role limitations because of physical health	24.8 (35.8)	56.4 (44.6)	29.5 (35.8)	53.8 (43.0)	0.35
SF-36 role limitations because of emotional problems	27.0 (40.1)	69.8 (42.4)	41.3 (39.9)	65.5 (42.6)	0.02
SF-36 energy	49.6 (13.1)	69.0 (20.3)	54.0 (12.0)	67.2 (20.6)	0.06
SF-36 emotional well-being	55.0 (12.5)	73.2 (19.7)	56.7 (12.4)	72.1 (20.1)	0.40
SF-36 social functioning	58.4 (22.8)	83.4 (17.6)	67.4 (20.0)	79.1 (21.0)	0.001
SF-36 pain	68.7 (22.4)	74.7 (24.1)	74.7 (21.8)	75.5 (23.5)	0.26
SF-36 general health	46.0 (13.6)	62.2 (24.6)	57.3 (11.6)	57.2 (21.8)	< 0.001
WHOQOL-BREF physical health	11.4 (2.2)	15.0 (2.8)	12.7 (1.9)	14.4 (2.6)	< 0.001
WHOQOL-BREF psychological health	10.7 (2.2)	14.2 (2.9)	11.6 (1.9)	13.7 (2.7)	0.002
WHOQOL-BREF social relationships	10.9 (2.3)	14.6 (2.3)	12.0 (2.2)	13.8 (2.3)	< 0.001
WHOQOL-BREF environmental health	11.5 (1.8)	15.2 (2.1)	12.5 (1.6)	14.4 (2.1)	< 0.001
Customer satisfaction scale	94.8 (14.0)	96.5 (10.7)	104.3 (10.4)	90.6 (9.3)	< 0.001
Comfort and cleanliness subscale	25.5 (4.5)	28.0 (3.1)	27.3 (3.7)	26.4 (3.2)	< 0.001
Nursing care subscale	32.5 (5.2)	31.2 (4.3)	36.0 (3.6)	29.5 (3.5)	< 0.001
Food services subscale	13.3 (3.4)	14.3 (2.5)	15.0 (2.8)	13.5 (2.2)	< 0.001
Facility care and services subscale	23.6 (3.7)	23.0 (3.2)	26.1 (2.9)	21.3 (2.7)	< 0.001

*Note:* WHOQOL-BREF, World Health Organization Quality of Life Scale.

Abbreviation: SD, standard deviation.

^a^The *p* value corresponds to the group × time interaction effect from the linear mixed model controlling for age, marital status, educational level, number of children, satisfaction with one's own financial situation, years of residing in this facility, activities of daily living, and instrumental activities of daily living (see Tables 6 and 7 for complete model details).

**Table 6:** Linear mixed-effects model results for resident outcomes (SF-36).

Parameter	Physical functioning		Role limitations (physical)		Role limitations (emotional)		Energy	
	Unadjusted	Adjusted	Unadjusted	Adjusted	Unadjusted	Adjusted	Unadjusted	Adjusted
*Fixed effects*								
Intercept	69.1 (62.8, 75.4)^∗∗∗^	84.8 (60.4, 109.2)^∗∗∗^	53.8 (45.3, 62.2)^∗∗∗^	80.2 (44.3, 116.2)^∗∗∗^	65.5 (56.8, 74.2)^∗∗∗^	76.5 (38.2, 114.8)^∗∗^	67.2 (63.7, 70.8)^∗∗∗^	81.0 (66.1, 95.9)^∗∗∗^
Time	0.7 (−0.8, 2.2)	−4.4 (−15.7, 6.9)	2.6 (0.6, 4.7)^∗∗^	2.6 (−8.3, 13.6)	4.3 (0.02, 8.5)^∗^	4.3 (−7.2, 15.7)	1.7 (0.8, 2.7)^∗∗∗^	1.7 (−2.8, 6.3)
Group	−45.1 (−53.7, −36.6)^∗∗^	0.7 (−6.9, 8.3)	−24.2 (−35.8, −12.7)^∗∗∗^	20.6 (1.1, 40.0)^∗^	−24.1 (−35.9, −12.2)^∗∗∗^	5.9 (−11.6, 23.3)	−13.1 (−18.0, −8.3)^∗∗∗^	7.0 (−1.1, 15.0)
Group × time	−2.0 (−4.0, 0.01)	−2.1 (−12.5, 8.4)	−7.4 (−10.2, −4.6)^∗∗∗^	−7.1 (−22.0, 7.9)	−18.7 (−24.5, −12.9)^∗∗∗^	−18.3 (−34.0, −2.7)^∗^	−6.2 (−7.5, −4.9)^∗∗∗^	−6.0 (−12.2, 0.2)
Age		0.1 (−0.2, 0.4)		0.2 (−0.2, 0.6)		0.2 (−0.2, 0.7)		0.1 (−0.1, 0.3)
Married		−7.4 (−13.9, −0.9)^∗^		−5.3 (−14.7, 4.0)		7.3 (−2.4, 17.1)		−0.1 (−3.9, 3.8)
University/college above		7.8 (−0.3, 15.8)		3.1 (−8.9, 15.2)		−2.8 (−15.1, 9.4)		−1.6 (−6.6, 3.3)
Senior high school		−0.2 (−7.4, 6.9)		−8.4 (−19.0, 2.2)		−8.9 (−19.8, 2.0)		−0.9 (−5.3, 3.5)
Junior high school		−1.8 (−10.0, 6.5)		−0.3 (−12.2, 11.6)		−3.9 (−16.3, 8.6)		0.9 (−4.0, 5.9)
Number of children		−4.9 (−12.3, 2.4)		−12.6 (−23.7, −1.6)^∗^		−13.9 (−25.3, −2.5)^∗^		−3.0 (−7.6, 1.6)
Financial satisfaction		19.3 (2.6, 36.0)^∗^		−16.9 (−41.1, 7.4)		2.7 (−22.5, 27.8)		−7.5 (−17.6, 2.5)
Years in facility		−0.5 (−1.2, 0.2)		−2.2 (−3.2, −1.1)^∗∗∗^		−1.5 (−2.6, −0.4)^∗∗^		−0.9 (−1.3, −0.4)^∗∗^
ADLs		−1.2 (−2.0, −0.4)^∗∗^		−1.0 (−2.2, 0.2)		−0.04 (−1.3, 1.2)		0.3 (−0.2, 0.8)
IADLs		−1.3 (−1.9, −0.7)^∗∗∗^		−1.0 (−1.9, −0.2)^∗^		−1.4 (−2.3, −0.5)^∗∗^		−1.0 (−1.4, −0.7)^∗∗∗^

*Random effects: covariance estimates*								
Resident within facility (intercept)	846.9	648.5	1527.6	1340.4	1465.3	1318.2	267.8	230.6
Residual	23.9	24.0	46.6	46.6	199.2	199.1	9.8	9.8

**Parameter**	**Emotional well-being**		**Social functioning**		**Pain**		**General health**	
	**Unadjusted**	**Adjusted**	**Unadjusted**	**Adjusted**	**Unadjusted**	**Adjusted**	**Unadjusted**	**Adjusted**

*Fixed effects*								
Intercept	72.1 (68.7, 75.6)^∗∗∗^	84.1 (69.6, 98.7)^∗∗∗^	79.1 (74.7, 83.4)^∗∗∗^	76.9 (56.7, 97.1)^∗∗∗^	75.5 (70.6, 80.3)^∗∗∗^	54.7 (32.8, 76.5)^∗∗∗^	57.2 (53.3, 61.1)^∗∗∗^	62.3 (44.9, 79.8)^∗∗∗^
Time	1.1 (0.5, 1.6)^∗∗∗^	1.1 (−3.5, 5.6)	4.4 (2.6, 6.2)^∗∗∗^	4.4 (−1.4, 10.1)	−0.8 (−2.9, 1.2)	−0.8 (−7.3, 5.7)	5.0 (3.4, 6.5)^∗∗∗^	5.0 (−0.1, 10.0)
Group	−15.4 (−20.1, −10.7)^∗∗∗^	−1.7 (−8.4, 5.1)	−11.7 (−17.7, −5.8)^∗∗∗^	6.6 (−4.3, 17.5)	−0.7 (−7.4, 5.9)	20.7 (8.4, 33.1)^∗∗^	0.2 (−5.1, 5.5)	20.6 (10.8, 30.4)^∗∗∗^
Group × time	−2.6 (−3.6, −2.1)^∗∗∗^	−2.7 (−8.9, 3.5)	−13.3 (−15.8, −10.9)^∗∗∗^	−13.2 (−21.1, −5.4)^∗∗^	−5.2 (−8.0, −2.4)^∗∗∗^	−5.0 (−13.9, 3.9)	−16.4 (−18.5, −14.3)^∗∗∗^	−16.2 (−23.0, −9.3)^∗∗∗^
Age		0.1 (−0.04, 0.3)		0.1 (−0.2, 0.3)		0.5 (0.2, 0.7)^∗∗^		0.1 (−0.1, 0.3)
Married		2.0 (−1.9, 5.8)		2.0 (−2.9, 7.0)		1.5 (−4.1, 7.0)		2.2 (−2.1, 6.6)
University/college above		−4.2 (−9.1, −0.6)		−2.7 (−9.3, 3.9)		−2.3 (−9.6, 5.0)		3.0 (−2.7, 8.8)
Senior high school		−1.6 (−5.9, 2.7)		0.3 (−5.4, 6.0)		2.4 (−4.0, 8.8)		−2.1 (−7.1, 2.9)
Junior high school		4.0 (−0.9, 8.9)		−0.04 (−6.4, 6.3)		0.6 (−6.5, 7.8)		0.8 (−4.7, 6.4)
Number of children		−4.0 (−8.4, 0.3)		−0.9 (−7.0, 5.2)		−9.3 (−16.1, −2.6)^∗∗^		−4.0 (−9.3, 1.3)
Financial satisfaction		−12.4 (−22.3, −2.4)^∗^		7.2 (−5.6, 20.1)		4.0 (−10.5, 18.5)		10.9 (−0.3, 22.2)
Years in facility		−0.8 (−1.2, −0.3)^∗∗^		−0.3 (−0.9, 0.2)		−1.3 (−1.9, −0.6)^∗∗^		−0.8 (−1.3, −0.3)^∗∗^
ADLs		0.2 (−0.3, 0.7)		0.3 (−0.4, 0.9)		0.3 (−0.4, 1.0)		0.5 (−0.1, 1.0)
IADLs		−0.8 (−1.1, −0.4)^∗∗∗^		−0.9 (−1.4, −0.5)^∗∗∗^		−0.8 (−1.3, −0.3)^∗∗^		−1.2 (−1.6, −0.8)^∗∗∗^

*Random effects: covariance estimates*								
Resident within facility (intercept)	262.3	235.9	384.5	360.0	477.8	457.8	308.2	276.9
Residual	3.6	3.6	35.5	35.5	46.2	46.3	26.4	26.4

*Note:* Values are parameter estimates with 95% confidence intervals in parentheses. Sample sizes: Number of facilities = 6; number of residents = 186; number of observations = 372 (186 residents × 2 time points).

Abbreviations: ADLs, activities of daily living; IADLs, instrumental activities of daily living.

^∗^ *p* < 0.05.

^∗∗^ *p* < 0.01.

^∗∗∗^ *p* < 0.001.

**Table 7:** Linear mixed-effects model results for resident quality of life and satisfaction outcomes.

Parameter	WHOQOL-BREF								Customer satisfaction	
	Physical health		Psychological health		Social relationships		Environmental health		Total score	
	Unadjusted	Adjusted	Unadjusted	Adjusted	Unadjusted	Adjusted	Unadjusted	Adjusted	Unadjusted	Adjusted
*Fixed effects*										
Intercept	14.4 (13.9, 14.9)^∗∗∗^	17.2 (15.3, 19.1)^∗∗∗^	13.7 (13.1, 14.2)^∗∗∗^	16.7 (14.8, 18.7)^∗∗∗^	13.8 (13.3, 14.3)^∗∗∗^	16.8 (14.8, 18.9)^∗∗∗^	14.4 (14.0, 14.8)^∗∗∗^	16.1 (14.5, 17.7)^∗∗∗^	90.6 (88.2, 93.0)^∗∗∗^	100.8 (90.2, 111.5)^∗∗∗^
Time	0.6 (0.4, 0.8)^∗∗∗^	0.6 (0.03, 1.2)^∗^	0.5 (0.3, 0.7)^∗∗∗^	0.5 (−0.1, 1.1)	0.8 (0.6, 1.0)^∗∗∗^	0.8 (0.2, 1.4)^∗∗^	0.8 (0.6, 0.9)^∗∗∗^	0.8 (0.3, 1.3)^∗∗^	5.9 (5.0, 6.7)^∗∗∗^	5.9 (2.7, 9.0)^∗∗^
Group	−1.8 (−2.5, −1.1)^∗∗∗^	1.6 (0.7, 2.5)^∗∗^	−2.1 (−2.8, −1.4)^∗∗∗^	1.1 (0.2, 2.0)^∗^	−1.8 (−2.5, −1.1)^∗∗∗^	0.4 (−0.6, 1.5)	−1.9 (−2.4, −1.3)^∗∗∗^	0.4 (−0.3, 1.1)	13.7 (10.4, 17.0)^∗∗∗^	21.0 (16.1, 25.8)^∗∗∗^
Group × time	−1.9 (−2.1, −1.7)^∗∗∗^	−1.9 (−2.7, −1.1)^∗∗∗^	−1.4 (−1.6, −1.2)^∗∗∗^	−1.4 (−2.2, −0.6)^∗∗^	−1.9 (−2.2, −1.6)^∗∗∗^	−1.9 (−2.7, −1.1)^∗∗∗^	−1.8 (−2.0, −1.6)^∗∗∗^	−1.8 (−2.5, −1.2)^∗∗∗^	−15.4 (−16.6, −14.3)^∗∗∗^	−15.3 (−19.6, −11.0)^∗∗∗^
Age		0.01 (−0.01, 0.03)		0.01 (−0.01, 0.03)		0 (−0.02, 0.03)		0.02 (0, 0.03)		0.04 (−0.1, 0.2)
Married		0.01 (−0.5, 0.5)		0 (−0.5, 0.5)		−0.2 (−0.7, 0.3)		−0.4 (−0.8, 0.1)		−1.3 (−4.0, 1.4)
University/college above		0.4 (−0.2, 1.0)		0.3 (−0.3, 1.0)		−0.2 (−0.8, 0.5)		0.3 (−0.2, 0.9)		0.2 (−3.2, 3.6)
Senior high school		0.1 (−0.4, 0.7)		0.1 (−0.5, 0.7)		−0.5 (−1.0, 0.1)		−0.1 (−0.6, 0.3)		−3.4 (−6.5, −0.4)^∗^
Junior high school		0.2 (−0.5, 0.8)		0.3 (−0.4, 0.9)		−0.7 (−1.3, −0.01)^∗^		0.4 (−0.1, 1.0)		1.5 (−2.0, 4.9)
Number of children		−0.3 (−0.9, 0.2)		−0.6 (−1.2, 0.02)		0.3 (−0.3, 0.9)		−0.2 (−0.7, 0.3)		−1.1 (−4.3, 2.1)
Financial satisfaction		−0.1 (−1.4, 1.2)		−1.5 (−2.8, −0.1)^∗^		−2.7 (−4.0, −1.3)^∗∗∗^		−1.7 (−2.8, −0.6)^∗∗^		−6.4 (−13.3, 0.6)
Years in facility		−0.1 (−0.2, −0.04)^∗∗^		−0.1 (−0.2, −0.1)^∗∗∗^		−0.01 (−0.1, 0.1)		−0.04 (−0.1, 0.01)		−0.3 (−0.5, 0.1)
ADLs		−0.1 (−0.1, 0.01)		0.02 (−0.1, 0.1)		0.02 (−0.1, 0.1)		−0.01 (−0.1, 0.04)		−0.1 (−0.4, 0.3)
IADLs		−0.2 (−0.2, −0.1)^∗∗∗^		−0.2 (−0.2, −0.1)^∗∗∗^		−0.1 (−0.2, −0.1)^∗∗∗^		−0.1 (−0.1, −0.03)^∗∗^		−0.2 (−0.4, 0.1)

*Random effects: covariance estimates*										
Resident within facility (intercept)	3.8	3.8	4.0	4.0	3.6	3.6	2.5	2.5	111.9	111.9
Residual	0.3	0.3	0.3	0.3	0.5	0.5	0.3	0.3	7.9	7.9

*Note:* Values are parameter estimates with 95% confidence intervals in parentheses. Sample sizes: Number of facilities = 6; number of residents = 186; number of observations = 372 (186 residents × 2 time points).

Abbreviations: ADLs, activities of daily living; IADLs, instrumental activities of daily living.

^∗^ *p* < 0.05.

^∗∗^ *p* < 0.01.

^∗∗∗^ *p* < 0.001.

## Data Availability

The data supporting the findings of this study are available from the corresponding author upon reasonable request.

## Supporting Information

Supporting Table 1. The flipped learning intervention.

Supporting Table 2. Interaction effects (group × time) for all subscales.

Supporting Figure 1. Changes in mean outcome scores of staff across time by experimental condition.

Note: Exp, experimental group; Control, control group; Time points: 1.0 = preintervention; 2.0 = postintervention.

Supporting Figure 2. Changes in SF-36 mean outcome scores of residents across time by experimental condition.

Note: Exp, experimental group; Control, control group; Time points: 1.0 = preintervention; 2.0 = postintervention.

Supporting Figure 3. Changes in mean outcome scores of quality of life and satisfaction of residents across time by experimental condition.

Note: Exp, experimental group; Control, control group; Time points: 1.0 = preintervention; 2.0 = postintervention.

## References

[1] Harvath T. A., Swafford K., Smith K. (2008). Enhancing Nursing Leadership in Long-Term Care. A Review of the Literature. *Research in Gerontological Nursing*.

[2] van Diepen C., Vestjens L., Nieboer A. P., Scheepers R. (2023). Nursing Home Staff Perceptions of Well-Being During the COVID-19 Pandemic: A Qualitative Study. *Journal of Advanced Nursing*.

[3] Feng Z., Glinskaya E., Chen H. (2020). Long-Term Care System for Older Adults in China: Policy Landscape, Challenges, and Future Prospects. *Lancet*.

[4] Perruchoud E., Weissbrodt R., Verloo H. (2021). The Impact of Nursing Staffs’ Working Conditions on the Quality of Care Received by Older Adults in Long-Term Residential Care Facilities: A Systematic Review of Interventional and Observational Studies. *Geriatrics*.

[5] Gandhi A., Yu H., Grabowski D. C. (2021). High Nursing Staff Turnover in Nursing Homes Offers Important Quality Information. *Health Affairs*.

[6] Li C., Shi C. (2022). Adverse Events and Risk Management in Residential Aged Care Facilities: A Cross-Sectional Study in Hunan, China. *Risk Management and Healthcare Policy*.

[7] Haunch K., Thompson C., Arthur A. (2021). Understanding the Staff Behaviours That Promote Quality for Older People Living in Long Term Care Facilities: A Realist Review. *International Journal of Nursing Studies*.

[8] Bourgeault I. L., Daly T., Aubrecht C., Armstrong P., Armstrong H., Braedley S. (2022). Leadership for Quality in Long-Term Care. *Healthcare Management Forum*.

[9] Zidén L., Erhag H. F., Wijk H. (2024). Person-Centered Care as a Tool to Reduce Behavioral and Psychological Symptoms in Older Adults With Dementia Living in Residential Care Facilities. *Geriatric Nursing*.

[10] Kim M., Beehr T. A. (2022). Empowering Leadership Improves Employees’ Positive Psychological States to Result in More Favorable Behaviors. *International Journal of Human Resource Management*.

[11] Kim M., Beehr T. A., Prewett M. S. (2018). Employee Responses to Empowering Leadership: A Meta-Analysis. *Journal of Leadership & Organizational Studies*.

[12] Tripathi N., Bharadwaja M. (2020). Empowering Leadership and Psychological Health: The Mediating Role of Psychological Empowerment. *Employee Responsibilities and Rights Journal*.

[13] Islam T., Zulfiqar I., Aftab H., Alkharabsheh O. H. M., Shahid M. K. (2024). Testing the Waters! the Role of Ethical Leadership Towards Innovative Work Behavior Through Psychosocial Well-Being and Perceived Organizational Support. *Journal of Organizational Change Management*.

[14] Al Otaibi S. M., Amin M., Winterton J., Bolt E. E. T., Cafferkey K. (2023). The Role of Empowering Leadership and Psychological Empowerment on Nurses’ Work Engagement and Affective Commitment. *International Journal of Organizational Analysis*.

[15] Amundsen S., Martinsen Ø. L. (2014). Empowering Leadership: Construct Clarification, Conceptualization, and Validation of a New Scale. *The Leadership Quarterly*.

[16] Lee A., Willis S., Tian A. W. (2018). Empowering Leadership: A Meta-Analytic Examination of Incremental Contribution, Mediation, and Moderation. *Journal of Organizational Behavior*.

[17] Khatoon A., Rehman S. U., Islam T., Ashraf Y. (2024). Knowledge Sharing Through Empowering Leadership: The Roles of Psychological Empowerment and Learning Goal Orientation. *Global Knowledge, Memory and Communication*.

[18] Kim M., Beehr T. A. (2020). The Long Reach of the Leader: Can Empowering Leadership at Work Result in Enriched Home Lives?. *Journal of Occupational Health Psychology*.

[19] Zhang X., Bartol K. M. (2010). Linking Empowering Leadership and Employee Creativity: The Influence of Psychological Empowerment, Intrinsic Motivation, and Creative Process Engagement. *Academy of Management Journal*.

[20] Cheong M., Spain S. M., Yammarino F. J., Yun S. (2016). Two Faces of Empowering Leadership: Enabling and Burdening. *The Leadership Quarterly*.

[21] Tuckey M. R., Bakker A. B., Dollard M. F. (2012). Empowering Leaders Optimize Working Conditions for Engagement: A Multilevel Study. *Journal of Occupational Health Psychology*.

[22] Backman A., Sjögren K., Lindkvist M., Lövheim H., Edvardsson D. (2017). Characteristics of Highly Rated Leadership in Nursing Homes Using Item Response Theory. *Journal of Advanced Nursing*.

[23] Cicolini G., Comparcini D., Simonetti V. (2014). Workplace Empowerment and Nurses’ Job Satisfaction: A Systematic Literature Review. *Journal of Nursing Management*.

[24] Spreitzer G., Sutcliffe K. M., Dutton J. E., Sonenshein S., Grant A. M. (2005). A Socially Embedded Model of Thriving at Work. *Organization Science*.

[25] Gilster S. D., Boltz M., Dalessandro J. L. (2018). Long-Term Care Workforce Issues: Practice Principles for Quality Dementia Care. *The Gerontologist*.

[26] Lacerenza C. N., Reyes D. L., Marlow S. L., Joseph D. L., Salas E. (2017). Leadership Training Design, Delivery, and Implementation: A Meta-Analysis. *Journal of Applied Psychology*.

[27] Kang H. Y., Kim H. R. (2021). Impact of Blended Learning on Learning Outcomes in the Public Healthcare Education Course: A Review of Flipped Classroom With Team-Based Learning. *BMC Medical Education*.

[28] Vermeerbergen L., McDermott A. M., Benders J. (2021). Managers Shaping the Service Triangle: Navigating Resident and Worker Interests Through Work Design in Nursing Homes. *Work and Occupations*.

[29] MacPhee M., Dahinten V. S., Hejazi S. (2014). Testing the Effects of an Empowerment-Based Leadership Development Programme: Part 1-Leader Outcomes. *Journal of Nursing Management*.

[30] Dahinten V. S., Macphee M., Hejazi S. (2014). Testing the Effects of an Empowerment-Based Leadership Development Programme: Part 2—staff Outcomes. *Journal of Nursing Management*.

[31] Cougot B., Gillet N., Gauvin J. (2022). Impact of Empowering Leadership on Emotional Exhaustion: A Controlled Interventional Study in a Large French University Hospital Complex. *Journal of Nursing Management*.

[32] Martin S. L., Liao H., Campbell E. M. (2013). Directive Versus Empowering Leadership: A Field Experiment Comparing Impacts on Task Proficiency and Proactivity. *Academy of Management Journal*.

[33] Cummings G. G., Hewko S. J., Wang M., Wong C. A., Laschinger H. K. S., Estabrooks C. A. (2018). Impact of Managers’ Coaching Conversations on Staff Knowledge Use and Performance in Long-Term Care Settings. *Worldviews on Evidence-Based Nursing*.

[34] Haynes A. B., Haukoos J. S., Dimick J. B. (2021). TREND Reporting Guidelines for Nonrandomized/Quasi-Experimental Study Designs. *JAMA Surgery*.

[35] Yu Z., Yoon J. Y., Grau B. (2016). How Do Levels of Nursing Home Adjustment Differ by Length of Stay?. *International Journal of Nursing Practice*.

[36] Folstein M. F., Folstein S. E., McHugh P. R. (1975). Mini-Mental State. *Journal of Psychiatric Research*.

[37] Lowe K. B., Kroeck K. G., Sivasubramaniam N. (1996). Effectiveness Correlates of Transformational and Transactional Leadership: A Meta-Analytic Review of the MLQ Literature. *The Leadership Quarterly*.

[38] Herold D. M., Fedor D. B., Caldwell S., Liu Y. (2008). The Effects of Transformational and Change Leadership on Employees’ Commitment to a Change: A Multilevel Study. *Journal of Applied Psychology*.

[39] Téllez A., García C. H., Corral-Verdugo V. (2015). Effect Size, Confidence Intervals and Statistical Power in Psychological Research. *Psychology in Russia*.

[40] Cook B. G., Cook L., Therrien W. J. (2018). Group-Difference Effect Sizes: Gauging the Practical Importance of Findings From Group-Experimental Research. *Learning Disabilities Research & Practice*.

[41] Fleiss J. L. (2011). *Design and Analysis of Clinical Experiments*.

[42] Nielsen K., Randall R., Christensen K. B. (2010). Does Training Managers Enhance the Effects of Implementing Team-Working? A Longitudinal, Mixed Methods Field Study. *Human Relations*.

[43] Shirazi M., Emami A. H., Mirmoosavi S. J. (2016). The Effects of Intervention Based on Supportive Leadership Behaviour on Iranian Nursing Leadership Performance: A Randomized Controlled Trial. *Journal of Nursing Management*.

[44] Hui C. (1994). Effects of Leader Empowerment Behaviors and Followers’ Personal Control, Voice and Self-Efficacy on In-Role and Extra-Role Performance: An Extension and Empirical Test of Conger and Kanungo’s Empowerment Process Model.

[45] Laschinger H. K., Wong C., McMahon L., Kaufmann C. (1999). Leader Behavior Impact on Staff Nurse Empowerment, Job Tension, and Work Effectiveness. *The Journal of Nursing Administration: The Journal of Nursing Administration*.

[46] Yeh W. Y., Cheng Y., Chen M. J., Chiu A. W. H. (2008). Development and Validation of an Occupational Burnout Inventory. *Taiwan Public Health Association*.

[47] Liu C. H., Chang L. C., Li I. C., Liao J. Y., Lin H. I. (2006). The Impact of Organizational and Psychological Empowerment on Organizational Commitment and Job Satisfaction Among Primary Health Professionals. *Journal of Nursing Research*.

[48] Spreitzer G. M. (1995). Psychological Empowerment in the Workplace: Dimensions, Measurement, and Validation. *Academy of Management Journal*.

[49] Liao S. W. (1978). The Relationships Among Elementary School Principal Leadership, Teachers’ Personal Traits, and Teachers’ Job Satisfaction.

[50] Weiss D. J., Dawis R. V., England G. W., Lofquist L. H. (1967). *Manual for the Minnesota Satisfaction Questionnaire*.

[51] Elo A. L., Leppänen A., Jahkola A. (2003). Validity of a Single-Item Measure of Stress Symptoms. *Scandinavian Journal of Work, Environment & Health*.

[52] Houdmont J., Jachens L., Randall R., Hopson S., Nuttall S., Pamia S. (2019). What Does a Single-Item Measure of Job Stressfulness Assess?. *International Journal of Environmental Research and Public Health*.

[53] Lu J. F., Tseng H. M., Tsai Y. J. (2003). Assessment of Health-Related Quality of Life in Taiwan (I): Development and Psychometric Testing of SF-36 Taiwan Version. *Taiwan Journal of Public Health*.

[54] Yo K. P. (2002). Development and Applications of the WHOQOL-Taiwan Version. *Formosan Journal of Medicine*.

[55] Mostyn M. M., Race K. E., Seibert J. H., Johnson M. (2000). Quality Assessment in Nursing Home Facilities: Measuring Customer Satisfaction. *American Journal of Medical Quality*.

[56] Chung C. P., Chang H. H., Yang T. W. (2011). Applying Structure Equation Modeling to Validate the Nursing Care Quality Satisfaction Scale in Nursing Home. *Journal of Senior Citizens Service Management*.

[57] Chiu C. J., Li M. L., Chou C. Y. (2022). Trends and Biopsychosocial Correlates of Physical Disabilities Among Older Men and Women in Taiwan: Examination Based on ADL, IADL, Mobility, and Frailty. *BMC Geriatrics*.

[58] Labrague L. J. (2024). Relationship Between Transformational Leadership, Adverse Patient Events, and Nurse-Assessed Quality of Care in Emergency Units: The Mediating Role of Work Satisfaction. *Australasian Emergency Care*.

[59] Becker T. E., Atinc G., Breaugh J. A., Carlson K. D., Edwards J. R., Spector P. E. (2016). Statistical Control in Correlational Studies: 10 Essential Recommendations for Organizational Researchers. *Journal of Organizational Behavior*.

[60] Trinkoff A. M., Lerner N. B., Storr C. L., Han K., Johantgen M. E., Gartrell K. (2015). Leadership Education, Certification and Resident Outcomes in US Nursing Homes: Cross-Sectional Secondary Data Analysis. *International Journal of Nursing Studies*.

[61] Fuchs M., Rossen A., Weyh A. (2023). Why Do Workers Leave Geriatric Care, and Do They Come Back? A Timing of Events Study. *International Journal of Nursing Studies*.

[62] Ginsburg L. R., Easterbrook A., Massie A. (2024). Building a Program Theory of Implementation Using Process Evaluation of a Complex Quality Improvement Trial in Nursing Homes. *The Gerontologist*.

[63] O’Brien E., Reifsnyder J. (2023). More of a Good Thing: A Framework to Grow and Strengthen the PALTC Careforce. *Journal of the American Medical Directors Association*.

[64] Safapour E., Kermanshachi S., Taneja P. (2019). A Review of Nontraditional Teaching Methods: Flipped Classroom, Gamification, Case Study, Self-Learning, and Social Media. *Education Sciences*.

[65] Hsu Y. R. (2011). Work-Family Conflict and Job Satisfaction in Stressful Working Environments: the Moderating Roles of Perceived Supervisor Support and Internal Locus of Control. *International Journal of Manpower*.

[66] Berning M. J., Zhong Z., White E. M., Niznik J. D., Berry S. D. (2023). Retention and Resilience of Nursing Home Staff During the COVID-19 Pandemic: Voices From the Frontline. *Journal of the American Medical Directors Association*.

[67] Williams J. A. R., Collins J. E., Gandhi A. (2024). Can Better Leadership Reduce Nursing Home Staff Turnover?. *Journal of the American Medical Directors Association*.

[68] Lautizi M., Laschinger H. K., Ravazzolo S. (2009). Workplace Empowerment, Job Satisfaction and Job Stress Among Italian Mental Health Nurses: An Exploratory Study. *Journal of Nursing Management*.

[69] Hidasi J., Neszmélyi G. I. (2022). Chapter 7: Social and Cultural Aspects in Taiwan’s Economic Development With Special Focus on the Education System and Gender Equality. *Changing Trade and Investment Relations of the Taiwanese Economy*.

[70] Burgess J., Kim H. M., Porath B. R. (2024). The Importance of Autonomy and Performance Goals in Perceived Workload Among Behavioral Health Providers. *Psychiatric Services*.

[71] Wu K. F., Hu J. L., Chiou H. (2021). Degrees of Shortage and Uncovered Ratios for Long-Term Care in Taiwan’s Regions: Evidence From Dynamic DEA. *International Journal of Environmental Research and Public Health*.

[72] Rantz M., Ersek M. (2023). Care Delivery, Quality Measurement, and Quality Improvement in Nursing Homes: Issues and Recommendations From the National Academies’ Report on the Quality of Care in Nursing Homes. *Journal of the American Geriatrics Society*.

[73] Jerofke T., Weiss M., Yakusheva O. (2014). Patient Perceptions of Patient-Empowering Nurse Behaviours, Patient Activation and Functional Health Status in Postsurgical Patients With Life-Threatening Long-Term Illnesses. *Journal of Advanced Nursing*.

[74] Chao S. F. (2019). Does Geriatric Nursing Staff Burnout Predict Well-Being of LTC Residents?. *Geriatric Nursing*.

[75] Malak M. Z., Abu Safieh A. M. (2022). Association Between Work-Related Psychological Empowerment and Quality of Nursing Care Among Critical Care Nurses. *Journal of Nursing Management*.

[76] Ebrahimi Z., Patel H., Wijk H., Ekman I., Olaya-Contreras P. (2021). A Systematic Review on Implementation of Person-Centered Care Interventions for Older People in Out-of-Hospital Settings. *Geriatric Nursing*.

[77] Poey J. L., Hermer L., Cornelison L. (2017). Does Person-Centered Care Improve Residents’ Satisfaction With Nursing Home Quality?. *Journal of the American Medical Directors Association*.

[78] Murray W. C., Holmes M. R. (2021). Impacts of Employee Empowerment and Organizational Commitment on Workforce Sustainability. *Sustainability*.

[79] Biron C., Karanika-Murray M. (2014). Process Evaluation for Organizational Stress and Well-Being Interventions: Implications for Theory, Method, and Practice. *International Journal of Stress Management*.

