# Supplementary material for: Impact of Empowering Leadership Training Through Flipped Learning Approach on the Well-Being of Nursing Staff and Residents in Long-Term Care Settings
Source: J Nurs Manag. 2025 Jul 28;2025:5815383. doi: 10.1155/jonm/5815383 (PMC12321434; doi:10.1155/jonm/5815383)
Supplement: Supporting Information — Additional supporting information can be found online in the Supporting Information section. [file 5815383.f1.docx]

| **Supplementary Table 1.** The flipped learning intervention | | |
| --- | --- | --- |
| **Topics and videos** | **Face-to-face activities** | **Post-class** **activities** |
| Introductions to flipped learning | Briefing, group discussion, and shared reflections | Reflections |
| Cultivating a mentoring culture and creating opportunities for facility development input | Briefing, group discussion, and case studies | Reflections |
| Facilitating care improvement contributions | Briefing, group discussion, and oral presentations | Reflections |
| Implementing new procedures for efficiency/outcomes | Briefing, group discussion, shared reflections, and oral presentations | Reflections |
| Encouraging ownership of professional development | Briefing, group discussion, case studies, shared reflections, and summary | Reflections |
| Developing an autonomy-promoting leadership style | Briefing, oral presentations, group discussion, and summary | Reflections |
| Improving nurse communication/ collaboration, and recognizing performance | Briefing, oral presentations, group discussion, and summary | Reflections |
| Involving nurses in decision-making | Briefing, group discussion, case studies, and practical exercises | Reflections |
| Providing feedback/coaching | Briefing, group discussion, case studies, and oral presentations | Reflections |
| Cultivating trust/respect | Briefing, group discussion, case studies, and practical exercises | Reflections |
| Supporting nurse well-being through policies /resources | Briefing, group discussion, case studies, and oral presentations | Reflections |
| Strengthening empowering leadership in long  -term care settings | Group discussion, shared reflections, and summary |  |

| **Supplementary Table 2**. Interaction effects (group × time) for all subscales | | | |
| --- | --- | --- | --- |
| Scale and subscale | Estimate (SE) | 95% CI | *p* value |
| **LEBS Subscales** |  |  |  |
| Enhancing meaningfulness of work | -7.6 (0.7) | -8.9, -6.2 | <0.001 |
| Fostering participation in decision-making | -5.7 (0.6) | -6.9, -4.5 | <0.001 |
| Facilitating goal accomplishment | -5.2 (0.4) | -6.0, -4.3 | <0.001 |
| Expressing confidence in high performance | -6.3 (0.7) | -7.6, -5.0 | <0.001 |
| Providing autonomy from constraints | -5.1 (0.5) | -6.0, -4.1 | <0.001 |
| **OBIS Subscales** |  |  |  |
| Personal burnout | 5.2 (0.4) | 4.3, 6.1 | <0.001 |
| Work-related burnout | 4.7 (0.4) | 3.9, 5.4 | <0.001 |
| Client-related burnout | 3.5 (0.5) | 2.6, 4.5 | <0.001 |
| Over-commitment to work | 5.0 (0.5) | 4.0, 5.9 | <0.001 |
| **SES Subscales** |  |  |  |
| Meaning | -2.1 (0.3) | -2.6, -1.6 | <0.001 |
| Competence | -2.0 (0.3) | -2.5, -1.4 | <0.001 |
| Self-determination | -2.1 (0.3) | -2.7, -1.5 | <0.001 |
| Impact | -1.3 (0.2) | -1.8, -0.8 | <0.001 |
| **CSS Subscales** |  |  |  |
| Comfort and cleanliness | -3.5 (0.7) | -4. 9, -2.0 | <0.001 |
| Nursing care | -5.2 (0.8) | -6.8, -3.7 | <0.001 |
| Food services | -2.5 (0.5) | -3.5, -1.4 | <0.001 |
| Facility care and services | -4.1 (0.6) | -5.4, -2.9 | <0.001 |
| SE, standard error; CI, confidence interval; LEBS, Leader Empowerment Behavior Scale; OBIS, Occupational Burnout Inventory Scale; SES, Spreitzer's Empowerment Scale; CSS, Customer Satisfaction Scale. | | | |

| 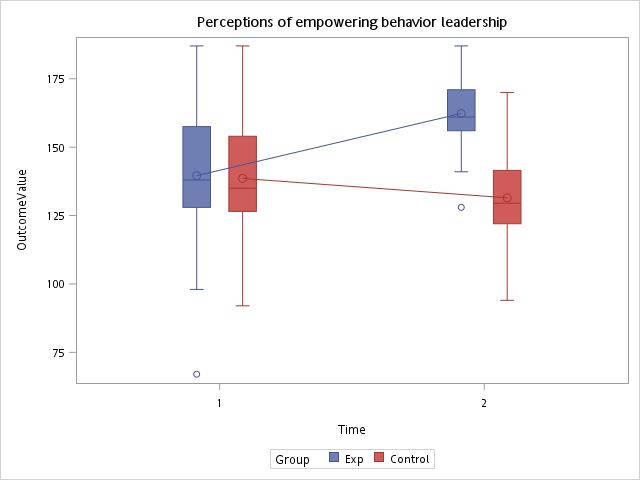 | 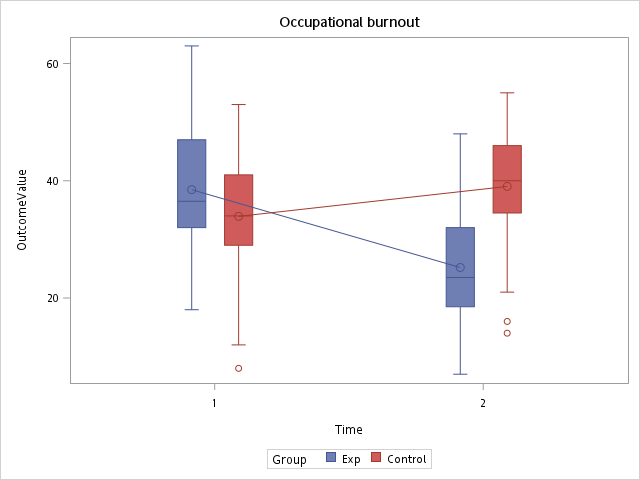 |
| --- | --- |
| 1. Perceptions of empowering behavior leadership | 1. Occupational burnout |
| 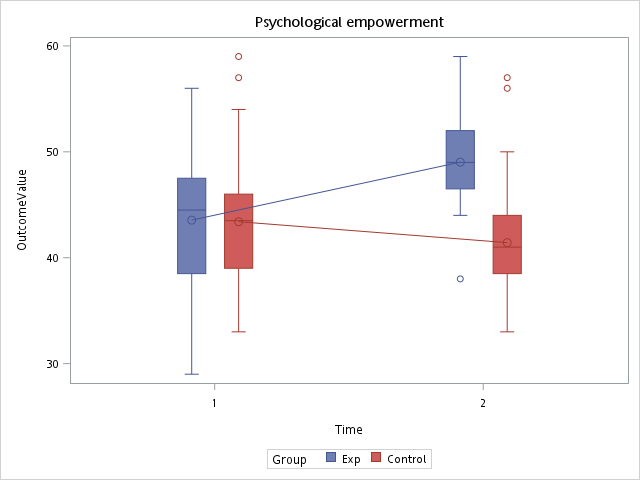 | 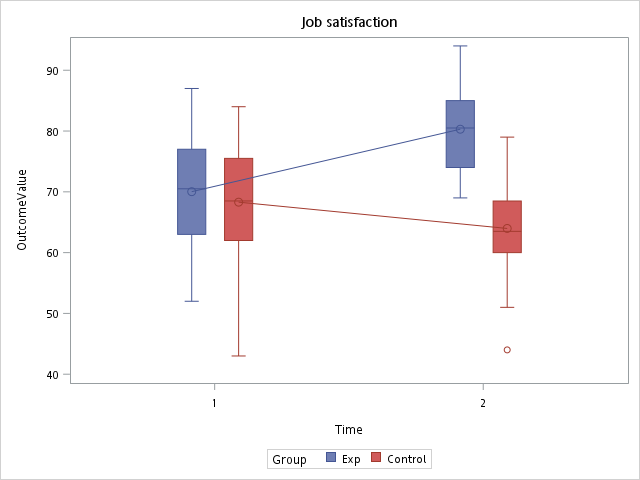 |
| C. Psychological empowerment | D. Job satisfaction |
| 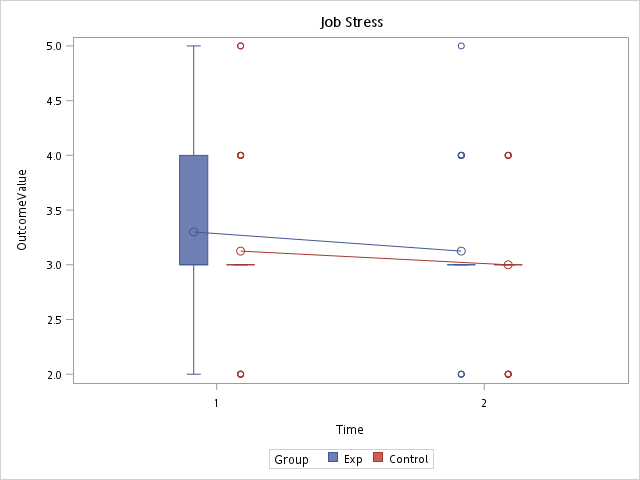 | |
| E. Job stress |  |
| **Supplementary Figure 1.** Changes in mean outcome scores of staff across time by experimental condition  Note: Exp, experimental group; Control, control group; Time points: 1.0 = pre-intervention; 2.0 = post-intervention. | |

| 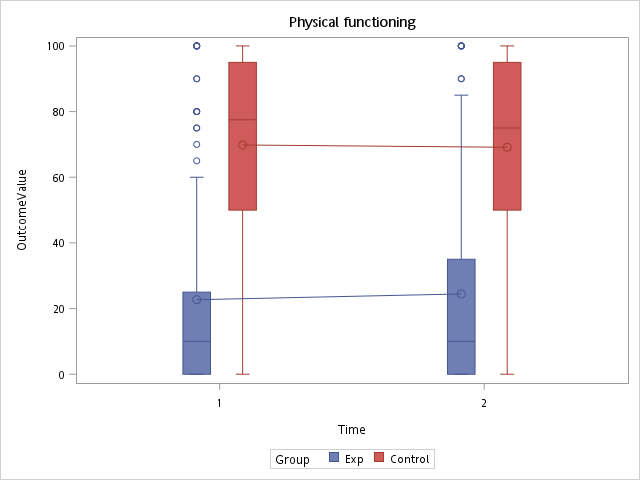 | 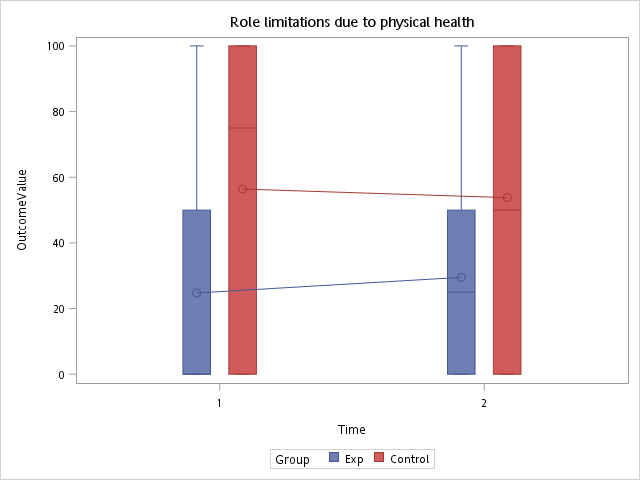 |
| --- | --- |
| A. Physical functioning | B. Role limitations due to physical health |
| 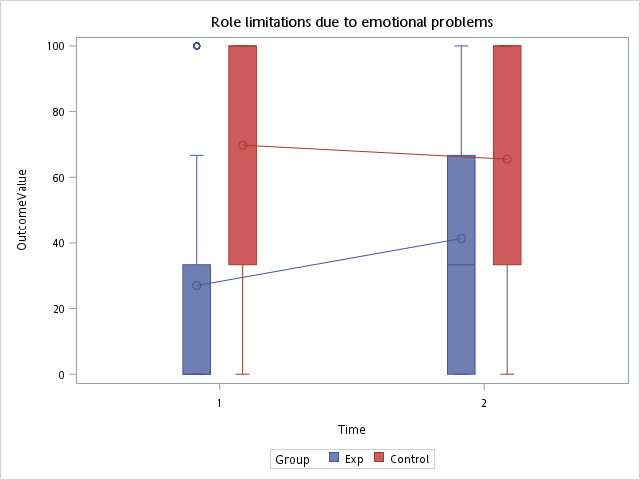 | 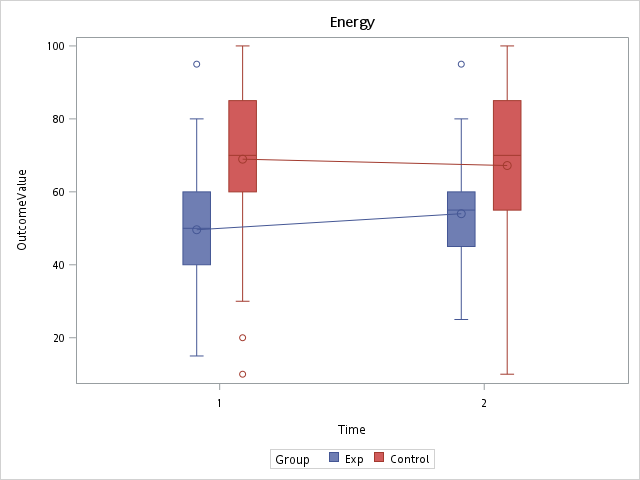 |
| C. Role limitations due to emotional problems | D. Energy |
| 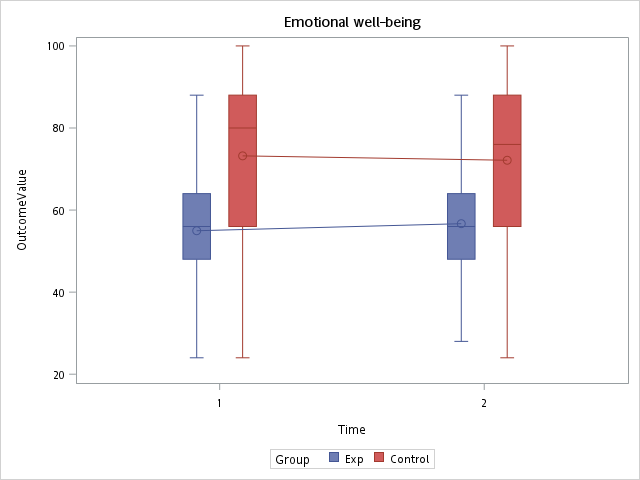 | 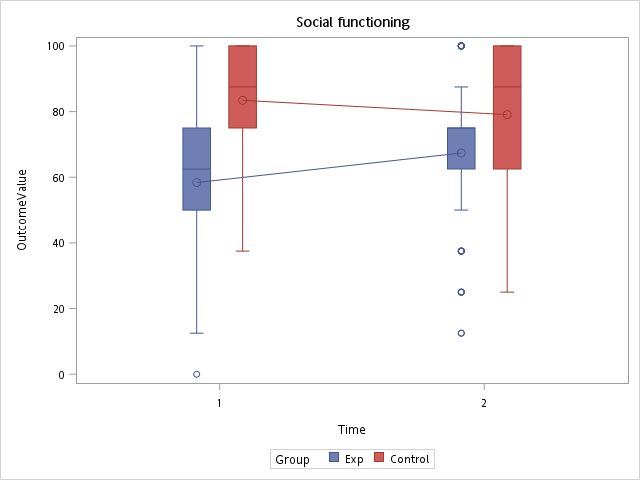 |
| E. Emotional well-being | F. Social functioning |
| 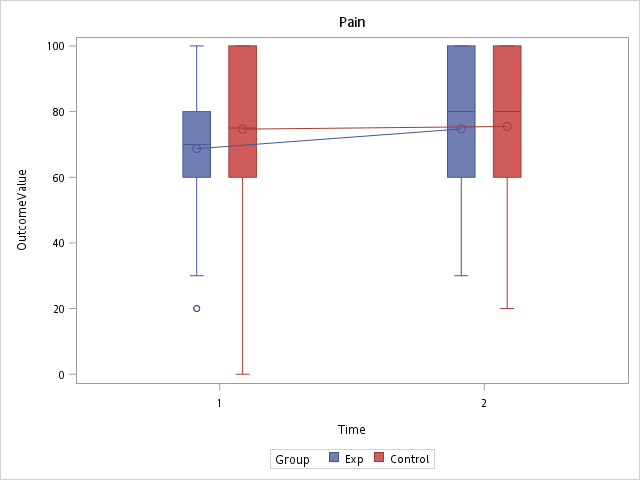 | 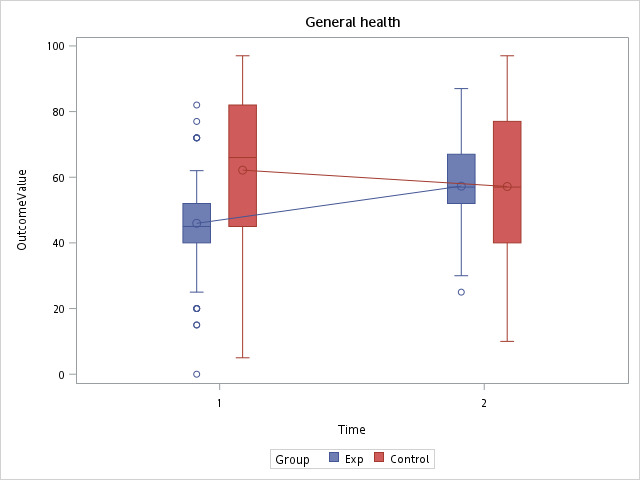 |
| G. Pain | H. General health |
| **Supplementary Figure 2.** Changes in SF-36 mean outcome scores of residents across time by experimental condition  Note: Exp, experimental group; Control, control group; Time points: 1.0 = pre-intervention; 2.0 = post-intervention. | |

| 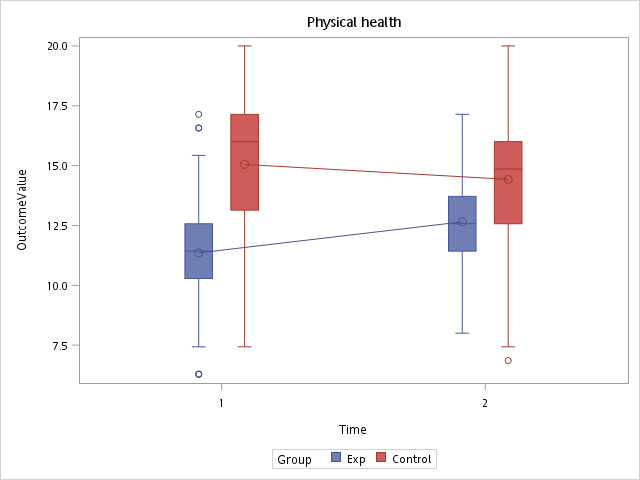 | 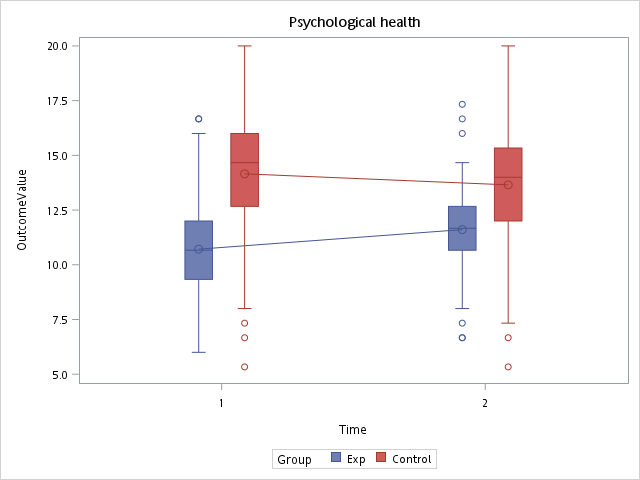 |
| --- | --- |
| A. WHOQOL-BREF physical health | B. WHOQOL-BREF psychological health |
| 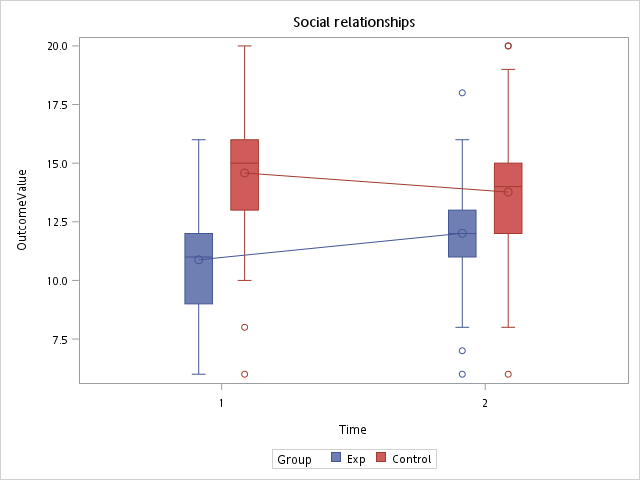 | 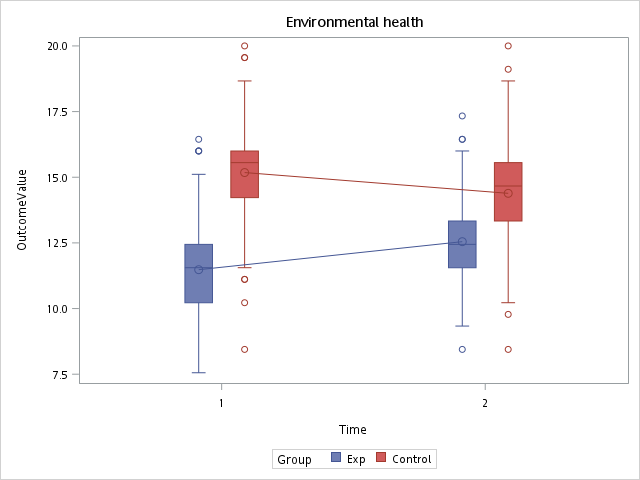 |
| C. WHOQOL-BREF social relationships | D. WHOQOL-BREF environmental health |
| 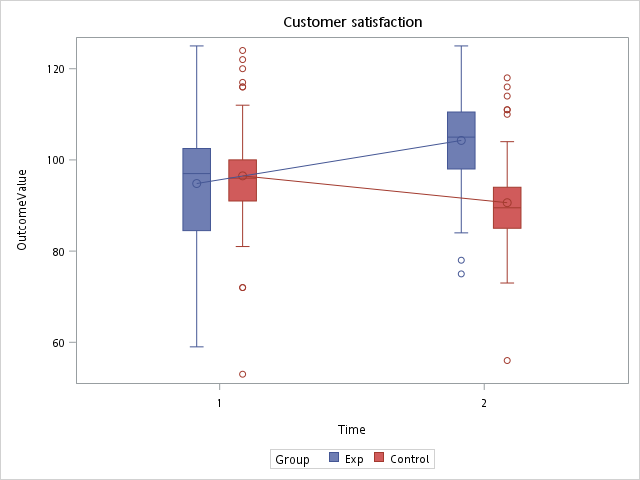 |  |
| E. Customer satisfaction |  |
| **Supplementary Figure 3.** Changes in mean outcome scores of quality of life and satisfaction of residents across time by experimental condition  Note: Exp, experimental group; Control, control group; Time points: 1.0 = pre-intervention; 2.0 = post-intervention. | |
